# Determination of Suitable Imaging Techniques for the Investigation of the Bonding Zones of Asphalt Layers

**DOI:** 10.3390/ma14247556

**Published:** 2021-12-09

**Authors:** Moritz Middendorf, Cristin Umbach, Stefan Böhm, Bernhard Middendorf

**Affiliations:** 1 Institute of Transport Infrastructure Engineering, Technical University of Darmstadt, 64287 Darmstadt, Germany; sboehm@vwb.tu-darmstadt.de; 2 Institute for Structural Engineering, Department of Structural Materials and Construction Chemistry, University of Kassel, 34127 Kassel, Germany; middendorf@uni-kassel.de

**Keywords:** layers bond, asphalt, bitumen emulsion, X-ray computer tomography, asphalt petrology

## Abstract

The material behavior of asphalt depends on its composition of aggregate, bitumen, and air voids. Asphalt pavements consist of multiple layers, making the interaction of the materials at the layer boundary important so that any stresses that occur can be relieved. The material behavior at the layer boundary is not yet understood in detail, as further methods of analysis are lacking in addition to mechanical methods. For this reason, the layer boundary of asphalt structures was analyzed using imaging methods. The aim of this research was to find an imaging method that allows a detailed analysis of the bonding zone of asphalt layers. Two different imaging techniques were used for this purpose. One is a 2-D imaging technique (asphalt petrology) and the other is a 3-D imaging technique (high-resolution computed tomography). Image analysis is a widely used technique in materials science that allows to analyze the material behavior and their composition. In this research, attention was paid to the analysis of the position of the bitumen emulsion, because the contained bitumen is supposed to bond the layers together. It was found that the application of 2-D imaging (asphalt petrology) lacked the precision for a detailed analysis of the individual materials at the layer boundary. With high-resolution computed tomography, a detailed view is possible to visualize the individual materials at the layer boundary in 3D. However, it is difficult to differentiate the materials because there are no gradations in the gray values due to the identical densities. However, it is possible to differentiate between the bitumen from the asphalt and from the emulsion if a high-density tracer is added to the bitumen emulsion for the CT studies. The results of the investigations are presented in this article.

## 1. Introduction

Many scientific questions about the material behavior of building materials can be answered by imaging methods in addition to the mechanical test methods. The material behavior of asphalt depends on the interaction between the constituents of the aggregate, the binder, and the air voids. In general, asphalt constructions are paved in several layers. Therefore, the interaction of the materials at the boundaries of the asphalt layers is particularly important so that any occurring stresses can be dissipated and do not lead to damage in the asphalt system. The effect of the layer bond depends on the interlocking of the aggregates, the bonding of the binder [1], and the friction between the aggregates [2,3] of the two layers. The factors of bonding, interlocking, and friction depend to varying degrees on the asphalt characteristics, the surface condition, the paving conditions, and the traffic load [1].

Due to the influence of heavy vehicles, high traffic volume, and adverse weather conditions, the service life of asphalt pavements can be reduced [4,5,6] because, for example, the layer bond is lacking. Krutcheva, Collop, and Thom [7] used FEM modeling to investigate the effects of lack of bond between asphalt layers. The results show that especially the lack of bond between the binder and base courses leads to a minimization of the service life.

A good interlock is achieved when the aggregate of the upper layer penetrates into the lower layer. In the “hot on hot” paving method, full satisfactory interlocking is usually achieved because the two layers are placed immediately one after the other and compacted in a single work step. With the “hot on cold” paving method, less interlocking is achieved [8]. For this reason, the “hot on cold” paving method mainly involves the application of spraying agents (bitumen emulsions) on the base layer, so that the reduced interlock can be compensated by gluing the layers together. The quality of the bonding depends on the type and quantity of spray agent applied and on the condition of the base surface. Dust and other impurities such as water must be removed before and after the spraying process [1]. West et al. [9] demonstrate that the application rate of the emulsion is essential. According to his findings, a low application rate achieves higher strength. Mohammad et al. [10] on the other hand, could not find an optimum for the application rate in his study. Raposeiras et al. [11] on the other hand, defines an amount of 250 g/m^2^ to 500 g/m^2^ as suitable and describes that a low amount leads to low resistance and a higher amount leads to sliding of the layers. Galaviz-González et al. [12] demonstrated in their work that bonding increases over time, leading to an improvement in layer bonding. In addition, it was demonstrated that bitumen emulsions with low amounts of bitumen (50%, 55%) achieved similar shear resistances as bitumen emulsions with 60% bitumen content.

In addition to the application of the bitumen emulsion, other influencing factors are relevant for the layer bonding. In 2014, Jaskula [13] demonstrated the influence of compaction work by producing test specimens with different compaction equipment. The test specimens produced in this series of tests under laboratory conditions achieved the best bond at the interface. The worst bond was measured for specimens compacted with a static roller.

Zhang [14] indicates that shear strength is dependent on the void content of the mix. Asphalt types with a coarse aggregate structure or a high percentage of air voids, such as porous asphalt, reduce the shear strength at the interface.

The influence of moisture on the layer bond was demonstrated by Al-Qadi et al. [15]. It was found on laboratory specimens that moisture in the layer interface leads to a reduction in shear strength.

The material behavior due to the various factors influencing the layer bonding (application of bitumen emulsion, moisture, dirt) as well as the failure mechanism at the layer boundary is not yet known in detail, since no other suitable analysis methods are available besides the mechanical test methods. Nowadays, the standard is to define the suitability of the layer bond based on the shear test according to Leutner [16]. With the shear force determined, conclusions about the quality of the layer bonding can be made. In further scientific work, the layer bond of asphalt was determined using cyclic test methods [8]. However, based on these results, only limited conclusions can be drawn about the structural behavior of the bonded layer. A suitable tool for obtaining further structural information is image analysis. This can be used to analyze the material behavior at the layer boundary; particularly relevant features here are the air void content at the layer boundary, the position of the aggregate and the position of the spray agent in the system, which can be used to determine adhesions and interlocking of the components.

Image analysis is a widely used technique in material science, and asphalt has been characterized in many research studies using polished or thin sections. Tielmann and Hill [17] have shown that asphalt petrological investigations based on sections can be used for comprehensive structural analysis in the 2-D plane. Asphalt petrology provides a very good basis for assessing the air void structure or damage in the microstructure.

Another imaging technique is computer tomography (CT), which generates detailed 3-D images. These images can be used to evaluate the material structure. The first investigations using CT on asphalt specimens were carried out in Switzerland in 1973. Air voids with a diameter of up to approx. 2.0 mm were detected in mastic asphalt specimens [18]. In other publications, different aspects regarding the types and distributions of air voids are considered [19,20,21].

**Figure 1:**
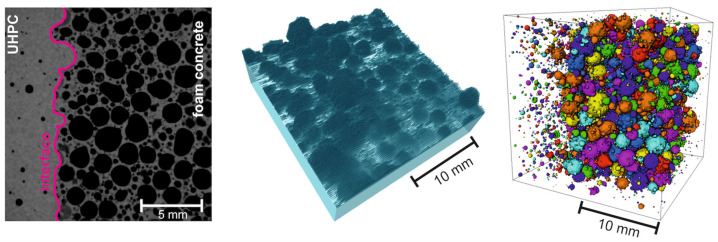
Visualization of the transition zones and pore space of different concretes using CT [22].

The imaging method of CT is becoming increasingly important for the understanding and further development of materials and is used in various other material technology issues, beside asphalt. Middendorf and Umbach [22] investigated the bonding area of different concretes using high-resolution computer tomography (µ-CT) and examined the interlocking of the composite zones (see Figure 1).

The following study was carried out in order to find a suitable method to enable a detailed analysis of the composite zone of asphalt specimens by means of imaging. In this study two imaging techniques of asphalt petrology and µ-CT were used.

## 2. Functioning of the Imaging Techniques Used

### 2.1. Asphalt Petrology

Asphalt petrology is a 2-D imaging technique that allows structural analysis of the aggregate, porosity, and binder components of asphalt in a plane using polished samples.

The method was introduced in Germany by Hart in 2015 and has since been continuously improved and further developed at the Technical University of Darmstadt [23].

**Figure 2:**
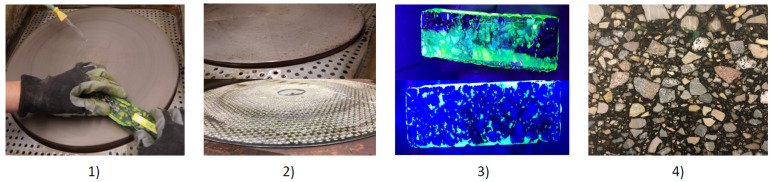
Grinding process of asphalt petrology: (**1**) water cooled grinder (**2**) different grinding wheels (**3**) specimen before and after grinding process (**4**) surface of specimen after grinding process (epoxy resin is only left in the voids). [17].

Using specially adapted preparation techniques for asphalt, sections are made from drill cores or laboratory specimens (e.g., Marshall specimens, plates from the rolling sector compactor). The sections should have a width of 100 to 150 mm to provide a representative sample of the structure. A saw with diamond-tipped blades is used to prepare the sections from the test specimens. During the cutting process, water is used as a cooling agent, as the binder in particular, tends to soften during cutting and can thus smear—filling air voids. After cutting, the specimen has to be dried. After drying the air voids are filled by vacuum impregnation with colored and fluorescent epoxy resin. The step is carried out in a vacuum chamber at a minimum pressure of 3 kPa. Any excess epoxy resin is polished off after complete curing (Figure 2), so that the epoxy resin is only present in the filled cavities.

**Figure 3:**
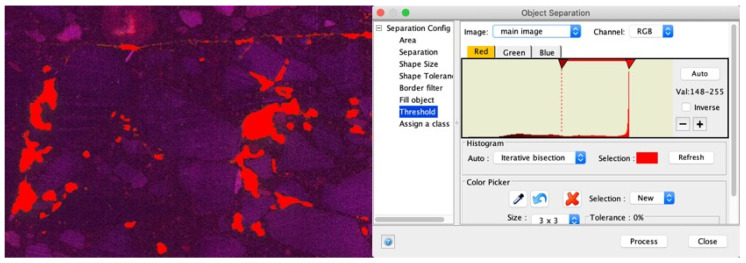
Identification of air voids using pixel-based segmentation.

The prepared surface of the polished sections is recorded as a UV scan by a flat board scanner. The generated UV scan is then analyzed with the JMicroVison software (Freeware, Version 1.3.3). The color values are assigned to the pixels; the threshold values set should distinguish between air voids, aggregate, and binder (Figure 3). After adjusting the threshold values, various algorithms are used to extract the individual objects from the entire object. Using digital image processing, all individual cavities are captured and stored in tabular form.

This data must then be processed further with other software tools (Matlab (Mathworks, Natick, MA, USA, Version R2021b), Octave (Freeware, Version 6.4.0), Excel (Microsoft, WA, USA, Version 16.55)), which allows, for example, to determine the absolute void content, the vertical, and horizontal void distribution and void orientation [24]. The different data types are listed in Table 1.

### 2.2. High-Resolution Computed Tomography (µ-CT)

**Figure 4:**
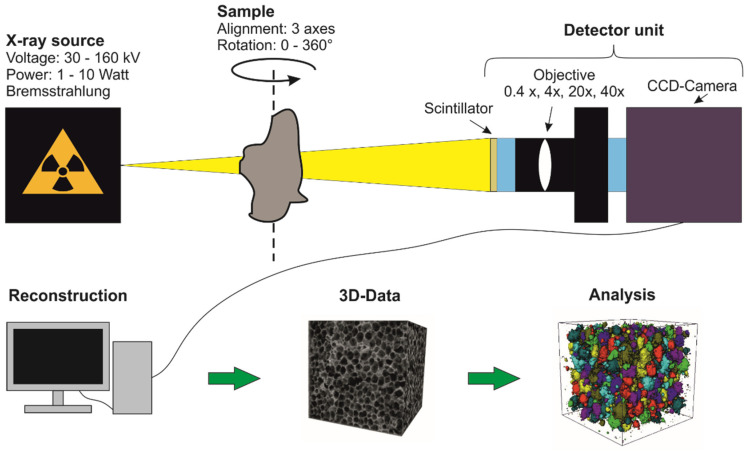
Schematic structure of a µ-CT measurement [22].

The increasing use of X-ray sources in medicine has also led to the development of high-energy X-ray sources for use in materials science. In order to be able to image the very fine and usually very complex structures with high resolution, so-called X-ray microscopy is often used, in which magnification objectives are used in the detector unit. A scintillation counter is mounted in front of each objective, which converts the X-rays into visible light that is recorded by a CCD camera (charge-coupled divice). Very high magnifications and resolutions can be achieved with this setup (Figure 4).

With µ-CT, single images are generated at different angles by radiating through the sample in different positions. A high radiation absorption of the sample, shows a dark CT signal; with a sample more permeable to radiation, the CT signal becomes brighter. The radiation absorption of a sample depends on various factors such as density, material thickness, and atomic number. The different gray values in a CT image therefore indicate different material densities. The images are acquired with 16 bit and therefore have gray values between 0 and 65535. The magnification of the image section to be viewed can be influenced by varying the projection geometry over the distance between the X-ray source, detector unit, and sample. To perform a 3-D reconstruction, images are taken in an angular range of at least 180°. To minimize artifacts during imaging, samples are usually rotated 360° during measurement. After the measurement, a 3-D image of the sample can be calculated from the individual projections (>1000) using a computer algorithm. The gray values are also inverted in this step, so that areas with a high density appear lighter while low densities are displayed darker.

**Figure 5:**
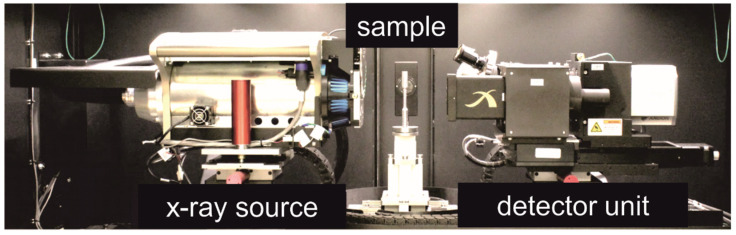
Measuring unit of the µ-CT Xradia 520 versa [22].

For the measurements, a high-resolution computer tomograph (µ-CT; see Figure 4 for functional principle) type Xradia 520 Versa, Carl Zeiss Microscopy GmbH (Oberkochen, Germany), was used, which has a spatial resolution of ≥0.7 µm. The measuring unit is shown in Figure 5. The X-ray source can be operated with a voltage between 30 and 160 kV at a power of 1 to 10 watts. With a total of 12 filters (6 low-emission and 6 high-emission filters), the X-ray beam can be adapted to the sample properties. A surface detector (magnification factor 0.4×) or three different objectives (4×, 20× and 40×) with scintillators can be used for signal detection. The sample stage can be moved on all three axes so that the region of interest (ROI) of the sample can be targeted. The reconstruction of the acquired projections is then performed automatically or manually with the XMReconstructor software (Zeiss Microscopy GmbH, Oberkochen, Germany, Version 11.1.8043.19515).

For sample preparation, the asphalt samples were sawed into prismatic specimens with a maximum length of 7 cm. The samples of bitumen emulsion with different amounts of tracer were placed between two glass slides. The measurement parameters are summarized in Table 2.

The data sets are evaluated using the Avizo 9.4 software from Thermo Fisher Scientific Inc. (Waltham, MA, USA). In addition to numerous display options, it offers various filters and segmentation options. By segmenting the individual projections as accurately as possible, models are created, for example of air inclusions in the microstructure or aggregate. These models can then be mathematically evaluated. In addition to various geometric quantities, position parameters and histograms can be generated and custom formulas can be included. In this way, material parameters, such as the aspect ratio of particles, can be output directly without having to calculate them in an additional work step.

For the following investigations, gray value histograms of partial areas of the images were evaluated and compared.

## 3. Investigation of the Layer Boundaries with Imaging Methods

In road construction, asphalt layers are paved either “hot on cold” or “hot on hot”. In the “hot on cold” paving method, the base is sprayed with bitumen emulsion to bond the two layers together before paving the superstructure. The amount of bitumen emulsion sprayed can vary depending on the type of base.

The aim of the first step of this study was to investigate a suitable method to observe the bonding zone of the asphalt layers and, in particular, to identifying the bitumen emulsion, thus enabling the position of the bitumen emulsion and the contact areas of the bitumen emulsion with the superstructure and the base to be analyzed. For this purpose, initial tactile tests were carried out using the presented imaging methods of asphalt petrology and µ-CT.

### 3.1. Results of the Investigation of the Layer Boundary by Asphalt Petrology

**Figure 6:**
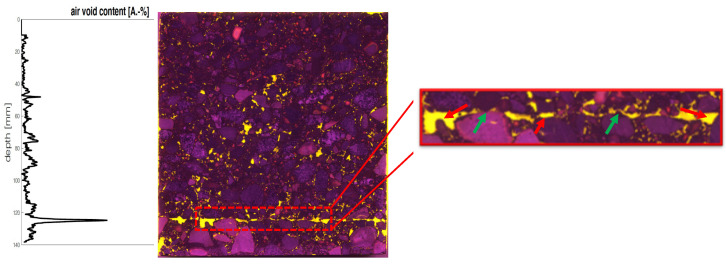
Analysis of voids over depth using asphalt petrology (red arrows: void analysis; green arrows: location of aggregate).

Using asphalt petrology, initial investigations were carried out on several drill cores. This showed that asphalt petrology is a suitable method for analyzing large asphalt samples. Initial findings on the distribution of air voids can thus be obtained. This allows the compaction of the asphalt layer to be assessed, which has an influence on the layer bond. Furthermore, a rough analysis of the layer boundary is possible. At the layer boundary, the void content, the position and type of voids, and the position of the aggregate can be assessed (see Figure 6 red and green arrows), which enable initial conclusions to be drawn about the material behavior at the layer boundary.

Asphalt petrology provides a 2-D insight into the sample, which is the basis for assessing the air void structure. The results show that although the asphalt petrology method allows deep insights, they are not sufficient in some cases, especially when identifying very thin (bitumen emulsion) and small components (air voids) in the asphalt structure. For a 3-D observation of a sample, several sections of a sample would have to be generated. This is possible in principle, but components of the sample are lost due to the 2 mm wide saw blade and thus falsify the results.

### 3.2. Results of the Layer Boundary Analysis by µ-CT

**Figure 7:**
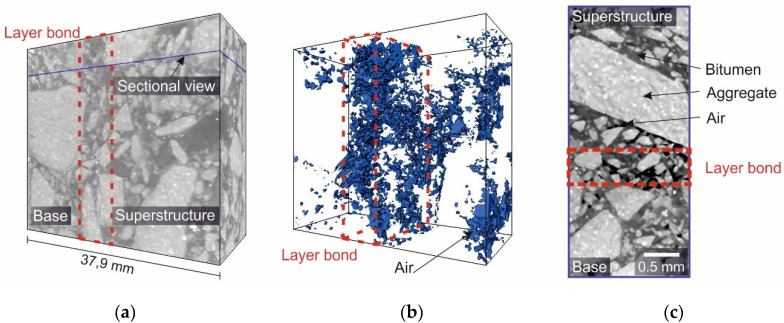
CT-Scan of an asphalt composite sample with a resolution of 42.9 µm: (**a**) 3-D reconstruction of the sample which provides an overview of the measurement; (**b**) model of the entrapped air; (**c**) sectional view into the sample.

Based on the results of the asphalt petrology studies, further investigations were conducted using a second imaging technique of computer tomography. Since different densities are detected in this method, the layer boundary could only be estimated by an accumulation of pores and aggregate boundaries. Figure 7 shows a sectional view of an asphalt sample consisting of a base and a superstructure. The two asphalt layers were bonded to each other via a bitumen emulsion. It can be seen that the aggregate (light gray) is bonded together by the bitumen (dark gray). In the area where the layers are bonded, a higher porosity (black) can be observed. However, the distribution and location of the bitumen emulsion cannot be differentiated.

## 4. CT Examinations with Tracer

The results have shown that the consideration of the composite zone in asphalt structures by asphalt petrology does not allow the bitumen emulsion to be identified. The results of the µ-CT showed that due to the identical density of the bitumen emulsion and the bitumen of the asphalt mixture (base; superstructure) of approximately 1.025 g/cm^3^, it is not possible to clearly observe the bitumen emulsion in particular in the scans. For this reason, in a second step of the study, an option was developed in which the bitumen emulsion in particular becomes visible.

In the scientific field as well as in medicine, the tracer method is well-known, in which a contrast agent of higher density (tracer) is added to the substance to be examined. Due to the difference in density, the area relevant to the investigation becomes visible and can then be analyzed. Castorena, Pape, and Mooney [25] added titanium dioxide as a powder to the binder before mixing it into the asphalt concrete in order to investigate the mixing between reclaimed asphalt and fresh asphalt. For this purpose, two mixtures with a high proportion of reclaimed asphalt were investigated using X-ray spectroscopy. Yang and Xu [26] also investigated the mixing between reclaimed asphalt and fresh asphalt using infrared analysis of an asphalt mix. The new asphalt was mixed with carboxy-terminated butadiene acrylonitrile (CTBN) as a tracer for identification. Five percent by weight has proven to be suitable.

### 4.1. Tracer Selection and Specimen Preparation

In the investigations carried out here barium sulphate (BaSO_4_, barite) was used as a tracer for the bitumen emulsion. The barite was added as a very finely milled powder as well as in the form of coarser barite sand. Both types are characterized by a significantly higher density (4.5 g/cm^3^) compared to bitumen (1.025 g/cm^3^) and to aggregate (in the range of 2.5 to 3.0 g/cm^3^). In addition, barite is chemically inert. The following figure shows the substances pictorially.

**Figure 8:**
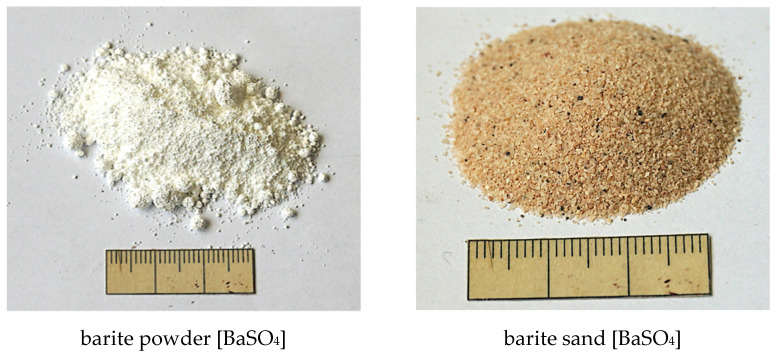
Tracers with different grain sizes.

**Figure 9:**
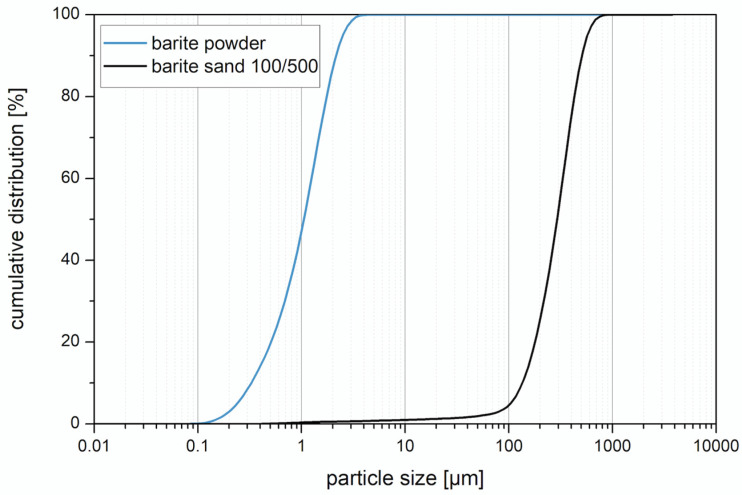
Particle size distribution of barite powder and barite sand.

The particle size distribution of the barite powder and barite sand (Figure 8) was measured by using a laser granulometer LS 13 320 XR from Beckman Coulter GmbH (Brea, CA, USA) using the wet mode with distilled water. The result was evaluated according to the Mie theory. The differences in particle size distribution can be seen in Figure 9. The barite sand has a significantly coarser particle size distribution compared to the barite powder.

**Figure 10:**
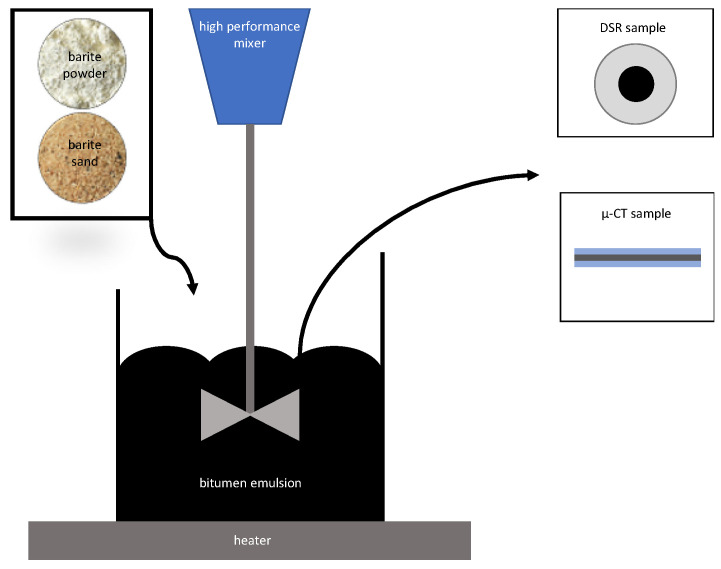
Schematic drawing of the material preparation.

To determine the suitability of barite as powder and as sand, the influence on the change of the binder properties was determined on the one hand and the visibility in the µ-CT on the other. For this purpose, the two tracers were added using a high-performance mixer (see Figure 10). The addition of the individual amounts of tracer (see Table 3) was carried out at a speed of 700 rpm. This ensured that the tracer was evenly distributed in the emulsion and that no agglomerates formed.

### 4.2. Effect of the Addition of Barite Sulfate on Imaging

**Figure 11:**
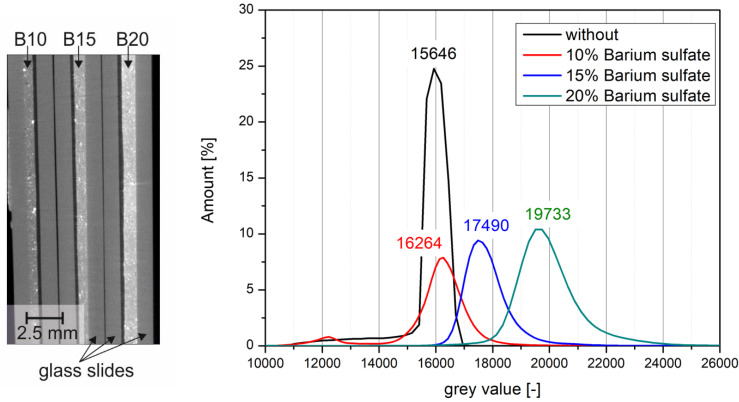
Cross-section through the CT scan of the bitumen samples with 10 wt%, 15 wt%, and 20 wt% barite powder (**left**) and evaluation of the gray scale histograms for the different dosages (**right**).

**Figure 12:**
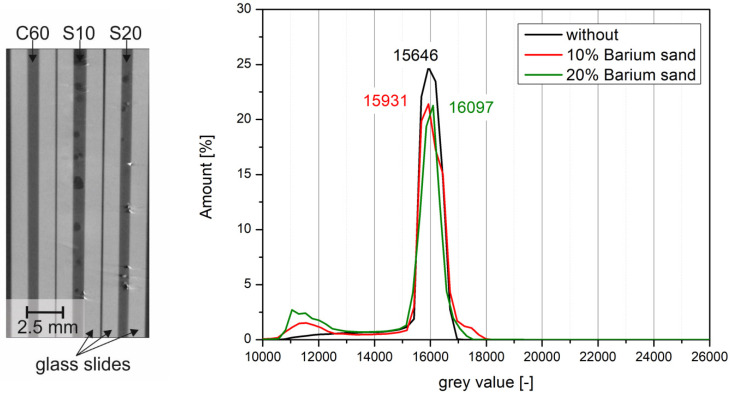
Cross-section through the CT scan of the bitumen samples without and with 10 wt% and 20 wt% barium sulfate sand (**left**) and evaluation of the gray scale histograms for the different dosages (**right**).

In order to quantify the influence of barite sulfate on the visibility of bitumen emulsion in CT scans, the samples were measured in the glass panes. For this purpose, the same gray scale definition was used for the minimum and maximum values in the reconstruction of the results. Subsequently, the gray values of the individual samples (Table 3) were analyzed (Figure 11 and Figure 12).

In the images, the bitumen without tracer (reference) was determined with a peak at a gray value of 15,646. By adding barite powder, the peaks became broader. The addition of 10 wt% barite powder resulted in a slight shift of the gray value to 16,264, but most of it still overlaps with the reference value. After addition of 15 wt%, the peak shifts more clearly into the lighter range and is at 17,490. Here, too, there is still a small overlap area. A complete separation of the peaks was achieved with the addition of 20 wt% barite powder; here the peak was shifted to 19,733.

**Figure 13:**
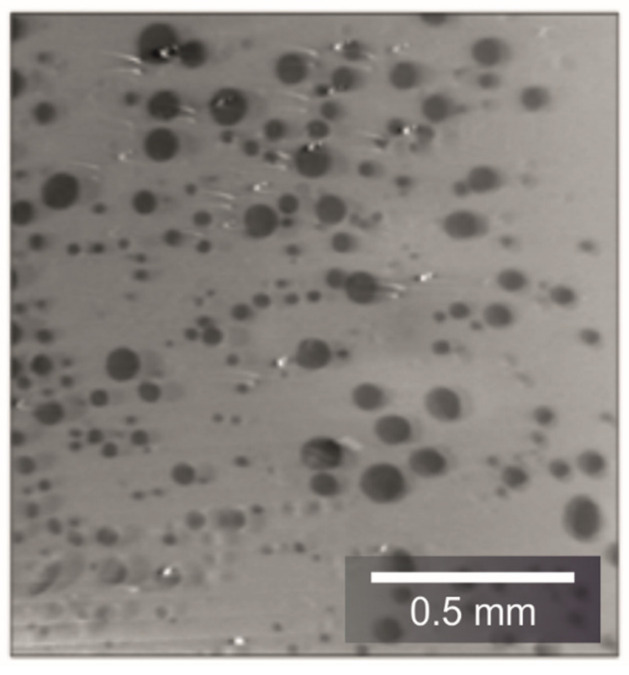
Sectional view through the bitumen emulsion with 10 wt% coarse barite sand.

The addition of barite sand resulted in very small shifts in the peaks from 15,646 (reference) to 15,931 (10 wt%) and 16,097 (20 wt%), respectively. All three peaks are directly above each other so that no distinction could be made between the bitumen emulsions. The air voids that were additionally included in the samples are recognizable (Figure 13). The bitumen emulsion without tracer as well as the one with barium sulfate powder are free of porosity.

### 4.3. Effect of the Addition of Barium Sulphate on the Binder Properties

In addition to the criterion of imaging with the µ-CT, the influence on the change of the binder properties with the addition of the tracers was investigated. For this purpose, the manufactured test specimens were examined with the dynamic shear rheometer (DSR) in the Temp Sweep according to AL DSR testing (T-Sweep). The test specimens were tested under defined oscillating stress and temperature range. The temperature was increased from 30 to 90 °C. During the test procedure, the complex shear modulus [Pa] and the phase shift angle [°] were determined and subsequently used to evaluate the influence of the addition of the tracer on the binder properties. The phase angle expresses the elastic recovery potential. Ideally elastic materials have a phase angle of 0° and react completely reversibly after the application of load has ended. Purely viscous materials react to the application of force with irreversible deformation. In this case, the phase angle is 90° [27]. The results are shown in the Black diagram. The Black diagram offers the advantage of displaying the measured rheological characteristics in one diagram.

**Figure 14:**
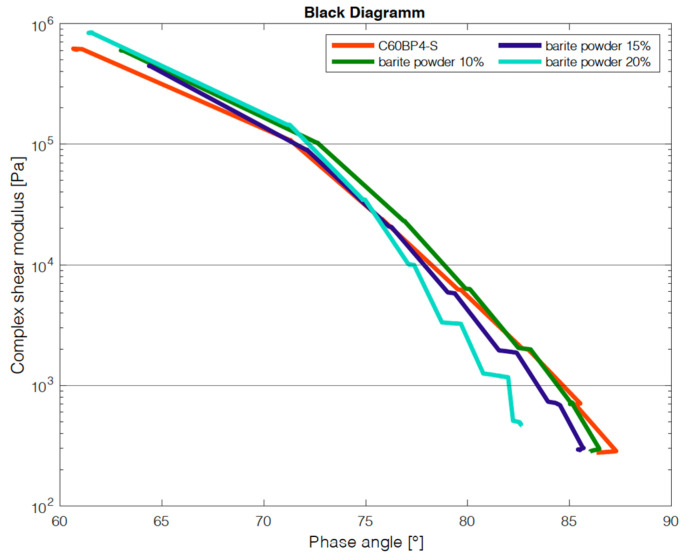
DSR results of addition of barite powder.

**Figure 15:**
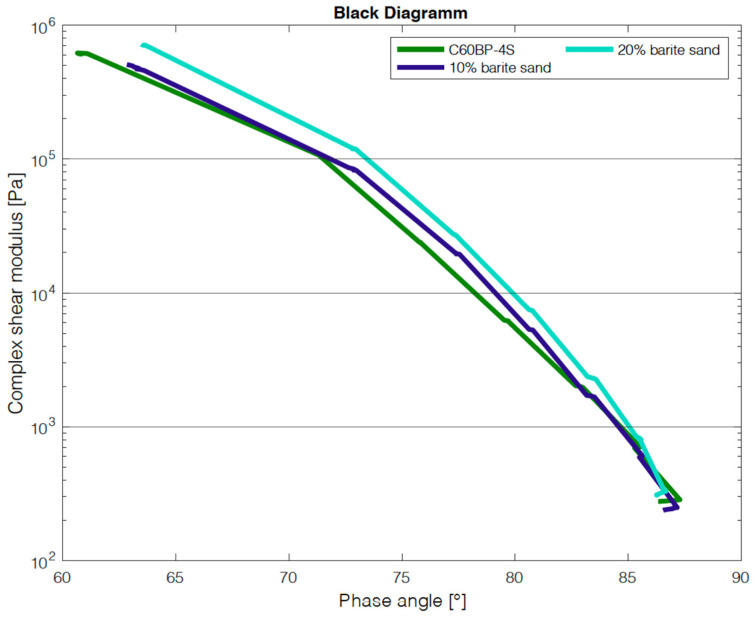
DSR results of addition of barite sand.

The results of the test with the DSR are shown in Figure 14 and Figure 15. Figure 14 shows the results from the test of adding the barium sulfate as powder. The addition of 10 wt% and 15 wt% does not cause much change in the complex shear modulus and phase angle compared to the results without barium sulfate (red line). The sample with 20 wt% addition shows a lower phase angle to the other samples. This indicates that at addition levels between 15 wt% and 20 wt%, the barite powder has a stiffening effect on the emulsion.

The results of adding barite sand are shown in Figure 15. It is striking that the addition of barite sand does not cause a great change compared to the addition of barite powder. At a small phase angle, the sample with 20 wt% barite sand has a higher shear modulus, but this equalizes again in the course of the test.

## 5. Discussion

Investigations using imaging methods have shown that with µ-CT an observation of the layer composite is possible at high resolution. However, when using µ-CT, a tracer must be added to observe the layer boundary of asphalt construction, otherwise the differentiation between the individual layers and the bitumen emulsion in between will not be possible due to its comparable densities. The results with the two tracers will be considered here, so that a suitable tracer and an appropriate amount of addition can be analyzed. For the evaluation, the gray values were compared with the phase angle and complex shear modulus at 40, 60, and 90 °C.

**Figure 16:**
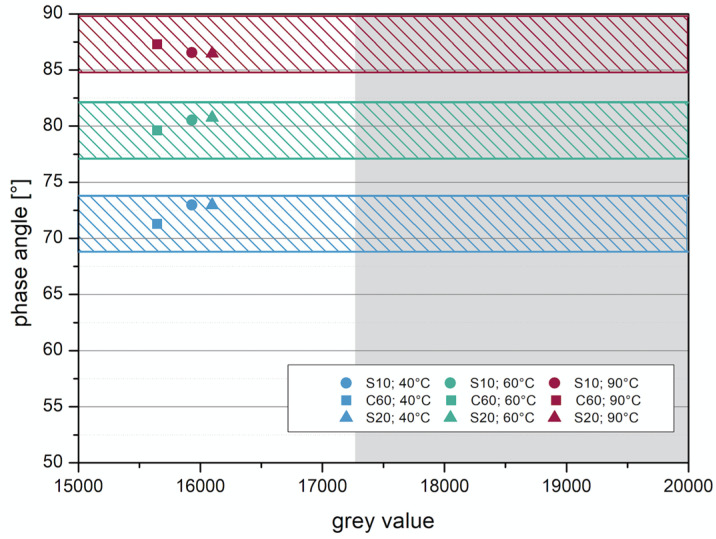
Correlation between phase angle and gray value—barite sand.

The addition of barite as tracer in the form of coarse-grained sand proved to be unsuitable for the observation with µ-CT. Although the investigations with DSR have shown that the changes in the properties due to the addition are only slight, the gray value, which is important for the evaluation of the µ-CT measurements, changes only very slightly. In Figure 16, the gray values are compared with the phase angle. It can be clearly seen that the addition of barite as sand has no great influence on the change in the required gray value. For a distinction, the gray value for the bitumen emulsion would have to be at least 17,250. Besides the ranges of the gray value, the changes of the properties should remain low, for this reason the colored ranges are shown. Inside these ranges, a property change is considered acceptable.

In addition, the barite added as sand forms air voids in the mixture (Figure 13), which falsifies the analysis. One explanation for this effect could be that the coarse-grained barite, due to its higher density compared to bitumen, sinks downwards following gravity during the cooling process, leading to the formation of voids. Similar segregation problems are already known from asphalt.

**Figure 17:**
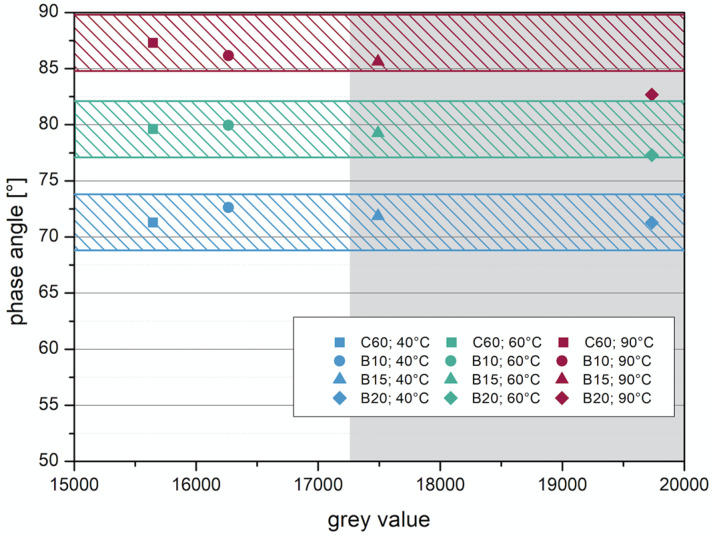
Correlation between phase angle and gray value—barite powder.

The use of barite powder, on the other hand, has proven to be useful. Here, the segregation problems have not occurred because the barite powder is very finely milled. In addition, the added barite powder achieves significant changes in the gray value, resulting in a distinction between the materials in the imaging. In Figure 17, the phase angle is plotted against the gray value. It can be clearly seen that the addition values of 15 and 20 wt% exceed the required gray value of 17,250. It is also clear from the figure that the phase angle decreases with the addition of 20 wt%.

**Figure 18:**
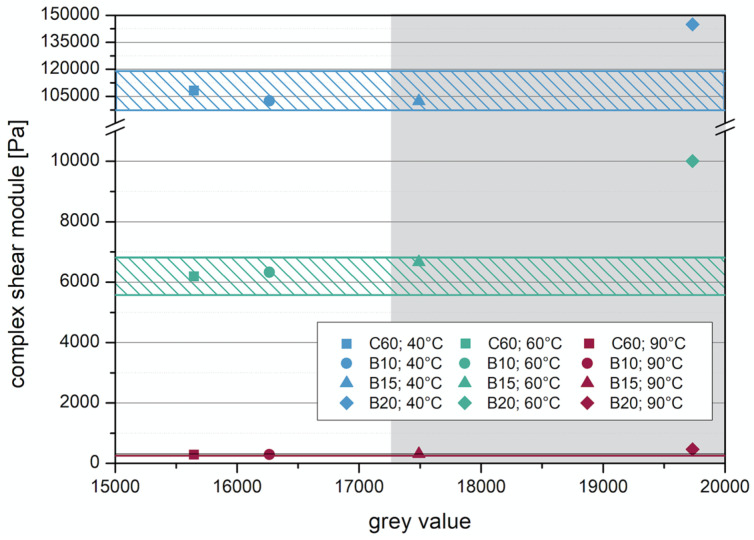
Correlation between complex shear module and gray value—barite powder.

It can also be seen in Figure 18, where the complex shear modulus is compared with the gray value. With an addition of 20 wt% barite powder, the complex shear modulus increases significantly compared to the addition of 10 and 15 wt% as well as the reference material, which can be interpreted as a stiffening of the material. The value at an addition of 20 wt% is outside the tolerance range, which was defined (colored shaded area).

Based on the analysis of the results obtained with µ-CT for imaging and DSR to assess the change in properties, it is clear that barium sulfate finely milled in powder form is suitable as a tracer. For imaging, the amount added must produces a significant change in the gray level. This change was obtained in this study with the addition amounts of 15 and 20 wt%. Considering the change in the properties of the bitumen emulsion, an addition of 20 wt% is too high, because this addition amount has too large a stiffening effect on the bitumen emulsion. With an addition of 15 wt%, on the other hand, only a slight change in the properties is measured, which is still within the permissible range.

The results show that property changes in the bitumen emulsion can be observed by using barite powder. The extent to which these changes have an influence on the mechan-ical properties of the composite must be critically examined. Although barite powder be-haves inertly and is used in medicine as a contrast agent, for example in abdominal CT scans, its effect on bitumen emulsions is still unclear. The first tests show that barite also behaves inertly in bituminous masses and does not trigger any chemical reactions. The property changes are thus due to particle interactions with the emulsion.

## 6. Conclusions

In this study, the layer bonding of asphalt structures was investigated using two different imaging techniques. The aim was to identify a suitable imaging technique for the observation of the layer boundary. According to the results, the main conclusions can be stated as follows:

- The investigation of the layer boundary of asphalt layers with imaging techniques has shown that the analysis of the layer boundary by imaging techniques is possible and can be used to achieve further knowledge about the material behavior. The imaging techniques used are different in detail. Through the 2-D imaging method, large specimens can be examined quickly and easily. Through the method, the void content at the layer boundary as well as the position of larger rock fractions at the layer boundary can be analyzed. For a detailed observation of the layer boundary, especially the position of the bitumen emulsion, the method lacks precession in the microstructural range.
- With µ-CT, detailed 3-D views of the materials at the layer boundary are possible. If two materials with identical densities are examined (bitumen from asphalt and bitumen emulsion as a spray-on material), a tracer must be added for identification.
- Fine grained barium sulfate powder has proved suitable as a tracer. By adding this tracer, the gray value for the analysis can be changed adequately, taking into account the properties. In this study, 15 wt% was found to be the optimum addition value.

In general, contrasting the binding zone in CT is possible by using barite powder. A lower dosage would be desirable in order to have less influence on the properties of the bitumen emulsion and thus on the bonding effect of the bitumen emulsion and thus on the behavior at the layer boundary.

Further investigations on the influence of the addition of the tracer on the properties (viscosity, adhesion behavior, breaking behavior) of bitumen and bitumen emulsions are to be carried out. In addition, other tracers besides barium sulfate are to be investigated for the application described.

## Figures and Tables

**Table 1:** Data types from the evaluation.

Type	Details
Area	Expansion of the cut air void area
Perimeter	Length of the boundary
Length	Longest expansion along the mayor axis
Orientation	Angle between the principal axis of an air void and the horizontal
Width	Longest expansion along the minor axis
Equivalent circular diameter	Diameter of a circle with the same area
Feret diameter	Longest expansion along defined orientation
Coordinates of the varycenter or centroid	Arithmetic mean position of the air void pixels

**Table 2:** Measurement parameters of the µ-CT acquisitions.

Measurement Parameter	Bitumen Emulsion	Asphalt Sample
Source setting	140 kV; 10 watt	140 kV; 10 wat
Source filter	Air	HE 1
Source distance	50 mm	125 mm
Detector distance	85 mm	55 mm
Resolution	25.6 µm	48.0 µm
Optical Magnification	0.4×	0.4×
Exposure time	1 s	6 s

**Table 3:** Addition quantities of the tracers.

Bitumen Emulsion	Tracer	Addition Quantity of the Tracer [wt%]	Addition Quantity of the Bitumen Emulsion [wt%]	Sample Name
C60 BP4-S	barite powder	10	90	B10
C60 BP4-S	barite powder	15	85	B15
C60 BP4-S	barite powder	20	80	B20
C60 BP4-S	barite sand	10	90	S10
C60 BP4-S	barite sand	20	80	S20

## Data Availability

The data presented in this study are avaibable on request from the corresponding author.
